# Effect of Heat Input on Microstructure and Properties of Laser-Welded 316L/In601 Dissimilar Overlap Joints in High-Temperature Thermocouple

**DOI:** 10.3390/ma16227114

**Published:** 2023-11-10

**Authors:** Hao Wang, Shengbin Zhao, Guifeng Luo, Zilin Tang, Xiang Li, Wenyuan Lu, Mingdi Wang

**Affiliations:** School of Mechanical and Electrical Engineering, Soochow University, Suzhou 215000, China; 20215229116@stu.suda.edu.cn (H.W.); 20225229068@stu.suda.edu.cn (G.L.); 20225229056@stu.suda.edu.cn (Z.T.); 20225229090@stu.suda.edu.cn (X.L.); 20225229129@stu.suda.edu.cn (W.L.)

**Keywords:** dissimilar laser welding, austenitic stainless steels, nickel-based alloys, microstructure, mechanical properties

## Abstract

Heat input, a crucial factor in the optimization of high-temperature thermocouple laser welding, has a significant impact on the appearance and mechanical properties of dissimilar welded joints involving stainless-steel- and nickel-based alloys. This study focuses on laser overlay welding of austenitic stainless steels and nickel-based alloys. The findings indicate that an increase in heat input has a more pronounced effect on the penetration depth and dilution rate. Under high heat input, the weld has cracks, spatter, and other defects. Additionally, considerable amounts of chromium (Cr) and nickel (Ni) elements are observed outside the grain near the crack, and their presence increases with higher heat input levels. Phase analysis reveals the presence of numerous Cr_2_Fe_14_C and Fe_3_Ni_2_ phases within the weld. The heat input increases to the range of 30–35 J/mm, and the weld changes from shear fracture to tensile fracture. In the center of the molten pool, the Vickers hardness is greater than that of the base metal, while in the fusion zone, the Vickers hardness is lower than that of the base metal. The overall hardness is in a downward trend with the increase of heat input, and the minimum hardness is only 159 HV_0.3_ at 40 J/mm. The heat input falls within the range of 28–30 J/mm, and the temperature shock resistance is at its peak.

## 1. Introduction

Austenitic stainless-steel- (316L) and nickel-based (Inconel 601) alloys are commonly employed as primary constituents in the manufacturing of high-temperature thermocouples for automotive engines owing to their exceptional capacity to withstand elevated temperatures and their favorable weldability properties [1]. In the context of laser-welding high-temperature thermocouple sensors, the combination of nickel-based alloys and stainless steel not only enhances corrosion resistance, oxidation resistance, and thermal conductivity of components but also lowers production expenses. Nevertheless, due to the differences in the microstructure and mechanical properties of dissimilar materials, the manufacture of such joints is extremely challenging.

International scholars have employed gas tungsten arc welding (GTAW) to join nickel-based alloys and stainless steel. For instance, researchers employed the gas tungsten arc welding (GTAW) technique to investigate the characteristics of butt-welded joints made from Inconel 617 and 310 alloys using various filler materials [2]. The study revealed that the mechanical properties of Inconel 617 filler material exhibited significant superiority. Researchers employed the same welding technique to investigate the properties of Inconel 718 with 310S joints [3] and found that the joints exhibited the highest tensile strength when filled with Inconel 625 material. Additionally, numerous international scholars employ electron beam welding as a technique for joining nickel-based alloys and stainless steel materials. For example, researchers utilized an electron beam to join Inconel 625 and SS304L butt joints [4]. It was observed that the weld exhibited a minor presence of microcracks, which can be attributed to the segregation of S elements within the molten pool. Researchers studied the microstructure of electron beam welding Inconel 617 and AISI 310 and found that the structure of the weld metal became finer with the increase in welding speed [5].

In contrast to the aforementioned welding techniques, laser welding exhibits reduced welding stress, less element segregation, and enhanced weld quality [6,7]. Additionally, it is capable of joining pre-fabricated precision components with minimal heat input and relatively minor part distortion. Researchers used carbon dioxide lasers to perform butt welding on Inconel 718 and AISI 416 at different speeds [8] and found that there was Nb-rich Laves phase segregation in the fusion region. Researchers employed fiber lasers to perform butt welding of Inconel 625 and DSS 2205 [9], varying the heat inputs. The findings indicate that significant intergranular segregation occurs under high heat input conditions, leading to the precipitation of numerous carbides. Conversely, the highest tensile strength is observed at low heat input levels. Researchers studied the effects of high power, high welding speed and low power, low welding speed on 316L/GH909 dissimilar metals laser welding joints under the same linear energy [10]. From the results, the weld with low power and low welding speed had a smaller temperature gradient and a lower cooling rate at the edge of the molten pool. In order to study the effect of welding process on the welding of nickel-based alloys and stainless steel, laser welding and argon arc welding were used for dissimilar metal welding [11]. From the results, the integral corrosion property is better than that of argon arc welded joint. However, the intergranular corrosion property of laser-welded joints is weaker than that of argon-arc-welded joints. The present study systematically examines the impact of post-weld heat treatment (PWHT) on the welded joints of Inconel 718 and 316L [12]. The findings indicate that the application of PWHT following welding leads to a notable reduction in residual stress within the welded joint. Additionally, PWHT has been observed to enhance the structural integrity, strengthen the synergistic effect between strength and plasticity, and improve the corrosion resistance of the welded joints.

The existing body of literature on the impact of heat input on the microstructure and properties of laser-welded 316L/In601 dissimilar overlap joints in high temperature thermocouples is currently limited. Therefore, this research paper aims to investigate the circumferential laser overlay welding process of Inconel 601 and 316L, with a specific focus on analyzing the resulting microstructure and mechanical properties.

## 2. Experimental Materials and Procedures

### 2.1. Material Preparation

**Figure 1:**
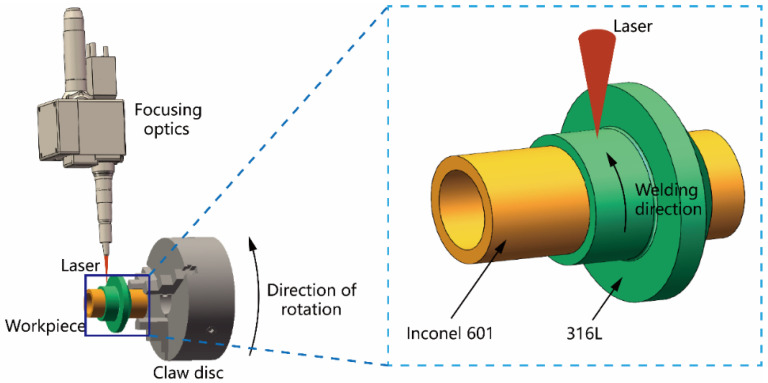
Schematic diagram of laser welding.

The focus of this research paper is on the process of laser overlay welding, specifically between a 1.00 mm wall thickness 316L casing and a 0.65 mm wall thickness Inconel 601 thermocouple. The light source employed in this study is a PB300CE solid-state pulsed laser with a maximum average output power of 300 W, an output laser wavelength of 1064 nm, and a fiber core diameter of 0.4 mm. The laser is positioned in a fixed manner directly above the central axis of the workpiece. The WJX125-57HS22-B model of a gapless rotary table is utilized to securely hold the workpiece by means of a three-jaw claw disc. This rotary table drives the workpiece to rotate, facilitating the completion of the circumferential welding process. The setup of the welding system is shown in Figure 1.

Before welding, the inner and outer walls of the casing and the outer wall of the thermocouple are wiped with alcohol to remove surface dust and oil. Pre-fixing of the 316L bushing and Inconel 601 thermocouple by riveting reduces contact surface clearance. The main chemical compositions of the two materials are shown in Table 1, expressed through weight percentages.

### 2.2. Experimental System

In the laser welding experiment, a total of five distinct heat inputs were employed, with the corresponding welding process parameters outlined in Table 2. Given the circumferential nature of the welding process, in order to mitigate the impact of arc craters on the weld’s performance, a time–power modulation strategy was implemented in the post-welding phase. The power decreases linearly over time to achieve a slow decrease in the input energy.

**Figure 2:**
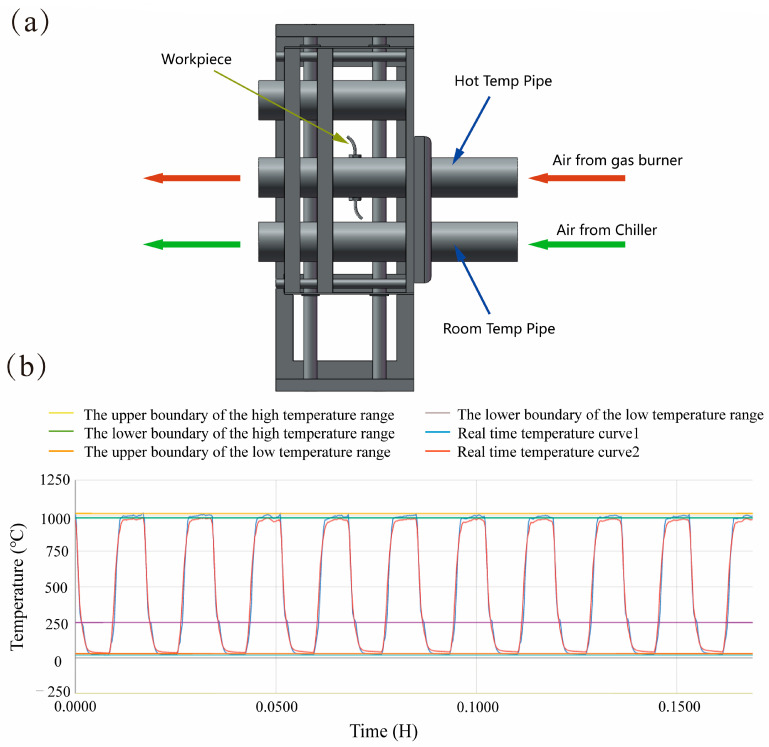
(**a**) Temperature shock test bench; (**b**) Temperature variation curve.

The installation of the product is required within the exhaust pipe of an automobile engine, whereby the welding seam will be subjected to the influence of alternating high- and low-temperature gases. Consequently, in the present investigation, following the completion of laser welding, the weldment undergoes a temperature impact test using a dedicated test stand, as depicted in Figure 2a. The test process is based on heating → high-temperature keeping → cooling → normal-temperature maintenance as a test cycle, with each cycle lasting for 60 s. The two gases used in the experiment were a high temperature gas at 1050 °C and a normal temperature gas at 50 °C. The gas was heated at a rate of 600 °C per second and cooled at a rate of 350 °C per second. The temperature change curve is shown in Figure 2b. To replicate the conditions of a welding seam service environment, a temperature impact test stand is employed for simulation purposes. The fracture failure time of the weld should be documented, and an assessment should be made regarding the weld’s capacity to endure temperature impact based on the duration of time.

### 2.3. Analysis Method

The specimens were subjected to axial cutting using an EDM wire cutting machine (DK7720). The weld cross-section was then ground and polished using SiC sandpaper (KAFUWELL, Hangzhou, China) and 0.5 μm diamond. To corrode the cross-section position of the weld, a mixed solution of HNO_3_ and HCl in a ratio of 1:3 was applied for a duration of 60 s. Subsequently, the specimens were immersed in an alcoholic solution and cleaned using ultrasonic waves with a frequency of 40 KHz. Afterward, the specimens were dried in a drying oven for a period of 12 h. The microstructure and energy spectrum scanning of the weld were observed using a field emission electron microscope (Regulus 8230, Carlsbad, CA, USA) equipped with an energy spectrometer. Additionally, the phase composition of the weld was analyzed using X-ray diffraction (Empyrean, Houston, TX, USA). The mechanical properties of the welds were evaluated using a tensile-shear machine (WDW-20Kn) at a constant speed of 3 mm/min. Vickers microhardness test was conducted on the weld cross-section, with a load of 300 g, a loading time of 15 s, and a spacing of 100 μm between each measurement point.

## 3. Results and Discussion

### 3.1. Macroscopic Morphology Characteristics

**Figure 3:**
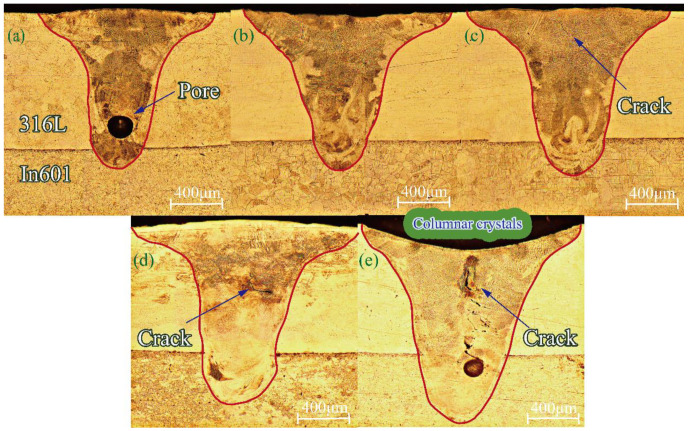
Optical microscopy images: (**a**) 25 J/mm; (**b**) 28 J/mm; (**c**) 30 J/mm; (**d**) 35 J/mm; (**e**) 40 J/mm.

In the realm of laser welding, conventional modes encompass heat conduction welding and keyhole welding. When the ratio of depth to width in a weld reaches 0.6, it falls under the category of penetration mode, which is considered one of the keyhole modes. However, in this particular mode, the stability of the locking hole is compromised, leading to an inability to sustain a continuous molten pool. The depth–width ratio in Figure 3 is measured to be 0.87, 0.78, 0.81, 0.84, and 0.92, respectively. These ratios indicate that the welding mode observed in the figure is keyhole welding. The results depicted in Figure 3 illustrate that the cross-sectional morphology of the weld assumes a “nail-shaped” appearance when the heat input falls within the range of 25–40 J/mm. Specifically, a heat input of 25 J/mm is capable of penetrating the contact thermal resistance between 316L and nickel-based 601, resulting in the formation of a molten pool with a depth of 0.15 mm in the nickel-based alloy. Although the weld surface is well-formed under these conditions, pores are observed within the weld pool. This occurrence is attributed to the rapid solidification of the molten pool due to the low heat input, which prevents the timely discharge of internal gas. Upon increasing the heat input to 30 J/mm, the depth of fusion expands to 0.27 mm, and the weld exhibits no obvious macro defects, such as porosity, cracks, biting edges, or collapse. However, when the heat input is further elevated to 35 J/mm, the depth of fusion continues to increase to 0.39 mm, and cracks emerge within the center of the weld. It is postulated that these cracks arise from elemental segregation in the center of the weld and the welding heat stress. Subsequently, at a heat input of 40 J/mm, the depth of fusion reaches 0.51 mm, accompanied by evident collapse on the weld surface and a significant increase in the number of cracks. This outcome is attributed to the substantial heat absorption and subsequent vaporization of the material, resulting in a strong backlash within the keyhole, metal splashing [13]. To mitigate the occurrence of element segregation caused by high heat input and excessively rapid solidification resulting from low heat input, a heat input of 28 J/mm is employed. It is observed that under these conditions, the weld exhibits no defects and forms a molten pool with a depth of 0.23 mm on the nickel-based alloy.

The composition and content of the elements present in the molten pool are primarily influenced by the proportion of the two base metals that are being melted. The ratio of the volume of the melted base metal to the total volume of the molten pool is referred to as the dilution rate. The dilution rate plays a significant role in the occurrence of microscopic elemental segregation within the weld. This, in combination with the cooling rate and welding thermal stresses, can potentially lead to the formation of solidification cracks in the weld [14]. In this study, the dilution rate is estimated by measuring the ratio of the melted area in the cross-section. The dilution rate of the Inconel 601 base metal over the entire weld metal is defined as DIN601.

**Figure 4:**
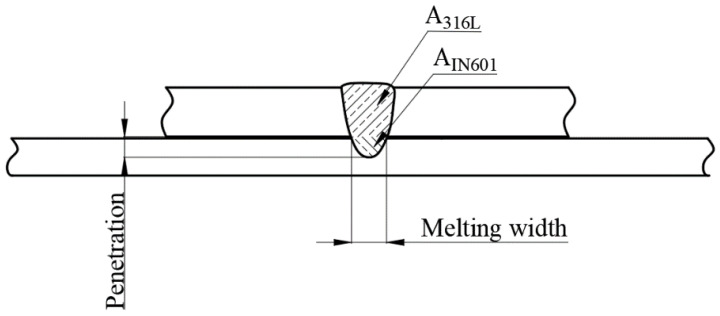
Schematic diagram of welded seam size measurement.

The measurement of the melting area of the two base materials was conducted using ImageJ software (NIH) in order to determine the weld dilution DIN601, as expressed in Equation (1). The melting area of the 316L base material is denoted as A316L, while the melting area of the Inconel 601 base material is denoted as AIN601. The melting width of Inconel 601 is defined as the width of the melted region, while the melting depth in Inconel 601 is defined as the depth of penetration, as illustrated in Figure 4.

(1) $$D_{IN601}=\frac{A_{IN601}}{A_{316L}+A_{IN601}}$$

**Figure 5:**
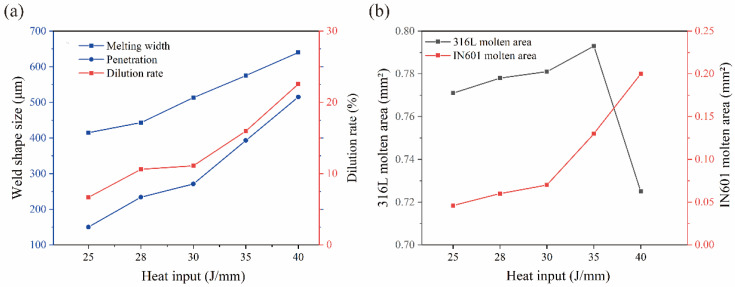
Effect of heat input on molten pool size: (**a**) penetration depth, penetration width, dilution rate; (**b**) molten area.

In Figure 5a, various cross-sections are measured with varying levels of heat input. It is observed that as the heat input increases, the dimensions of melting width, penetration depth, and dilution rate also increase. Specifically, when the heat input is raised from 25 J/mm to 40 J/mm, the penetration depth rises from 0.15 mm to 0.51 mm, the penetration width increases from 0.42 mm to 0.64 mm, and the dilution rate escalates from 6.6% to 22.6%. The width of the melting zone demonstrates a nearly linear relationship with the augmentation of heat input. When the heat input is 40 J/mm, the melting width surpasses 0.6 mm.

The melting area of the dissimilar material is depicted in Figure 5b. As the heat input is augmented, the material absorbs a greater amount of heat, resulting in an increased proportion of melted Inconel 601 base metal. Notably, the melting area experiences a more pronounced increase when the heat input surpasses 30 J/mm. Similarly, the melting area of 316L stainless steel also increases with the augmentation of heat input. However, once the heat input reaches 35 J/mm, the melting area of 316L stainless steel decreases significantly. This decline can be attributed to the phenomenon of liquid metal spattering in the molten pool when the heat input exceeds 35 J/mm. Nevertheless, the dilution rate remains unaffected and continues to exhibit a linear increase.

In summary, inadequate or excessive heat input can result in the formation of defects in the shape of a weld. Optimal weld formation is achieved when the heat input is maintained at 28 J/mm.

### 3.2. Microstructural Characteristics

**Figure 6:**
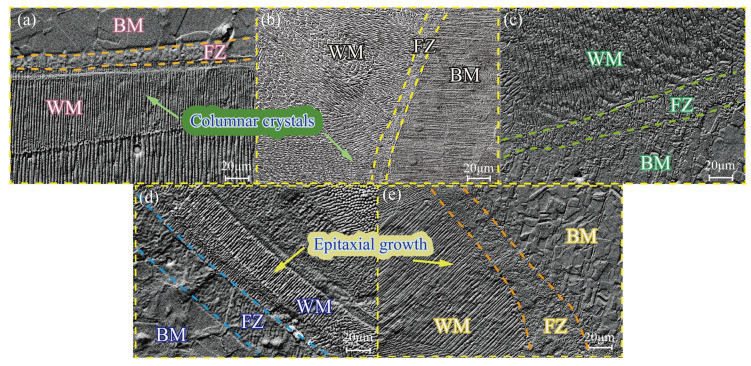
Scanning electron microscopy images (**a**) 25 J/mm; (**b**) 28 J/mm; (**c**) 30 J/mm; (**d**) 35 J/mm; (**e**) 40 J/mm.

Figure 6 depicts a microscopic image illustrating the fusion line under varying heat inputs. The image reveals the presence of numerous cellular crystals on the side of the fusion line adjacent to the molten pool. These cellular crystals grow perpendicularly towards the molten pool due to the reduction in temperature gradient at the initial stage of solidification, resulting in the formation of a subcooling zone. The stability of the planar crystal interface is compromised, resulting in the formation of numerous bulging spores that continue to expand towards the interior of the molten pool. Simultaneously, the solute is expelled beyond the sub-grain boundary, causing a decrease in temperature within the liquid phase surrounding the sub-grain boundary. This leads to the development of a substructure within the grain, with its primary axis direction remaining aligned with the growth direction. On the side of the fusion line adjacent to the base metal, a soft tissue region emerges. This area primarily exists in a semi-molten state during the welding process, as the liquid alloying elements fail to form austenite. This condition negatively impacts the performance and leads to the accumulation of impurities with low melting points [15]. The extent of the soft zones increases with higher heat inputs, which is contingent upon the magnitude of the temperature gradient near the fusion line. When the heat input is substantial, the effective melting point is rapidly attained, resulting in a small temperature gradient and slow cooling, thereby expanding the range of the soft zone.

**Figure 7:**
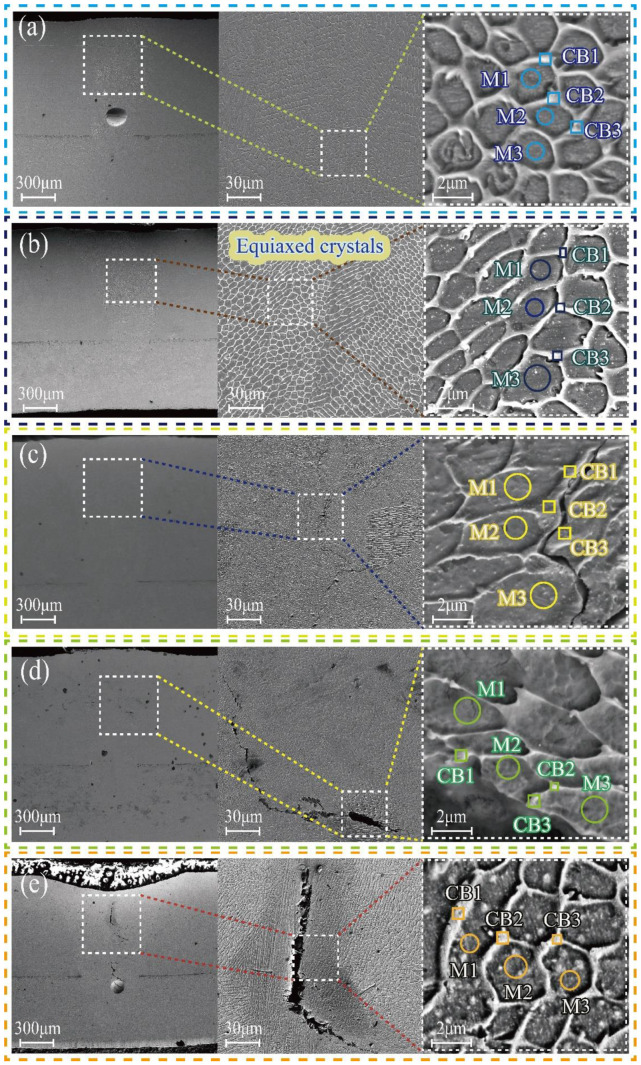
Scanning electron microscopy images: (**a**) 25 J/mm; (**b**) 28 J/mm; (**c**) 30 J/mm; (**d**) 35 J/mm; (**e**) 40 J/mm.

Figure 7 illustrates the microstructure of the fusion zone when subjected to varying heat inputs. It is observed that an equiaxial crystal structure is formed at the center of the weld, which is attributed to the rapid solidification characteristics of laser welding. The solidification process of the welding molten pool is influenced significantly by changes in component subcooling. As the solidification progresses, the temperature gradient in the middle of the molten pool decreases, leading to an increase in solidification speed and the expansion of the component supercooling region. Consequently, an equiaxed crystal structure is formed in the middle of the molten pool.

At a heat input of 25 J/mm, no cracks were observed in the weld. However, holes appeared in the lower middle part of the weld. This phenomenon occurs as a result of the rapid solidification of the melt pool during welding with low heat input. The keyhole inside the metal vapor and protective gas becomes blocked, leading to the formation of porosity. When the heat input was increased to 30 J/mm, 35 J/mm, and 40 J/mm, varying degrees of cracks were observed in the center of the weld. The size of these cracks increased with the increase in heat input. These cracks occur during the late stage of solidification in the molten pool, specifically in the solid–liquid state. They are known as solidification cracks and are formed within the brittle temperature range [16]. The formation of these cracks is a result of the combined effects of elemental segregation and thermal stress during welding. As the heat input increases, the time that the weld remains within the brittle temperature range also increases. Consequently, the susceptibility to solidification cracking is heightened [17]. Additionally, the increase in heat input leads to an increase in thermal stresses. These stresses exceed the stress extremes in the center of the weld, further enhancing the tendency for cracking [18]. From a microstructural perspective, it has been observed that when the heat input is set at 28 J/mm, the occurrence of pores and cracks can be reduced.

**Figure 8:**
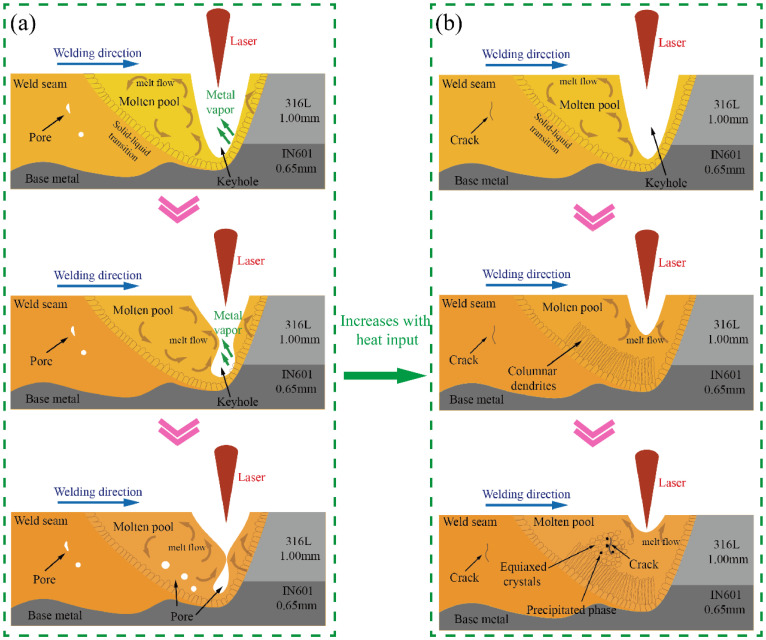
Schematic diagram of welding process: (**a**) stomatal schematic; (**b**) schematic diagram of cracks.

Figure 8 depicts a schematic diagram illustrating the welding process. In Figure 8a, the generation of stomata is demonstrated. When the heat input is low, it becomes challenging to maintain a balance between the pressure generated by the metal vapor and the gravity of the molten pool. Consequently, the keyhole wall collapses towards the center, trapping gas at the bottom of the keyhole and forming bubbles. These bubbles then rise to the surface of the molten pool due to buoyancy. However, the upper stainless steel solidifies rapidly due to its higher thermal conductivity compared to the underlying nickel-based alloy. As a result, the bubbles are unable to ascend and instead become pores [19,20]. Figure 8b illustrates the mechanism of crack formation. When the heat input is high, the molten pool solidifies at a slower rate, leading to the appearance of equiaxed crystals in the center of the weld. Additionally, precipitation phases form between the grains. As the temperature falls below the melting point, the cooling process becomes sluggish, causing the weld to remain in the “brittle temperature range” for an extended period. Consequently, under the influence of thermal stress, the plastic strain capacity is insufficient to counteract the strain generated during the solidification process, resulting in the formation of cracks.

The results of point scanning of the inside and outside of the grains at the center position and counting the elemental content inside and outside of the grains are shown in Table 3. The findings indicate that the primary elements present outside the grains are chromium, iron, and nickel. Moreover, the content of chromium and nickel outside the grains increases as the heat input increases. Although the iron content has decreased, it remains relatively high. In the solidification process of a molten pool under non-equilibrium conditions, the diffusion process occurring at the interface between the solid and liquid phases is hindered as a result of the rapid cooling rate. This phenomenon results in the enhancement of solute elements, such as carbon, chromium, and nickel, at the interfaces between grains. The solubility of chromium (Cr), nickel (Ni), and carbon (C) is relatively limited, leading to their tendency to segregate at the boundaries between grains. Due to the melting of nickel-based alloys into the molten pool, the Fe% gradually decreases as the melting volume of In601 increases. This suggests that the precipitates at the grain boundaries consist of phases rich in chromium, nickel, and iron. The variation in elemental content outside the grains at different heat inputs can be attributed to elemental segregation during non-equilibrium solidification, resulting in an increase in chromium and nickel content outside the grains and a decrease in iron content. It is worth noting that the analysis of carbon elements may have a significant error due to the inability of the energy spectrum to analyze elements with a nuclear charge less than 11. However, the relative content of carbon elements at different positions still provides some reference value. Table 3 reveals that the carbon content outside the grain is considerably higher than that inside the grain, and this difference increases with the rise in heat input.

The presence of elevated levels of carbon in the vicinity of the grain structure leads to the formation of numerous carbides along the grain boundaries, resulting in a depletion of chromium in these regions [21]. When subjected to residual stresses during welding, the chromium-deficient areas adjacent to the grain boundaries experience plastic deformation beyond their limit, leading to the formation of cracks.

**Figure 9:**
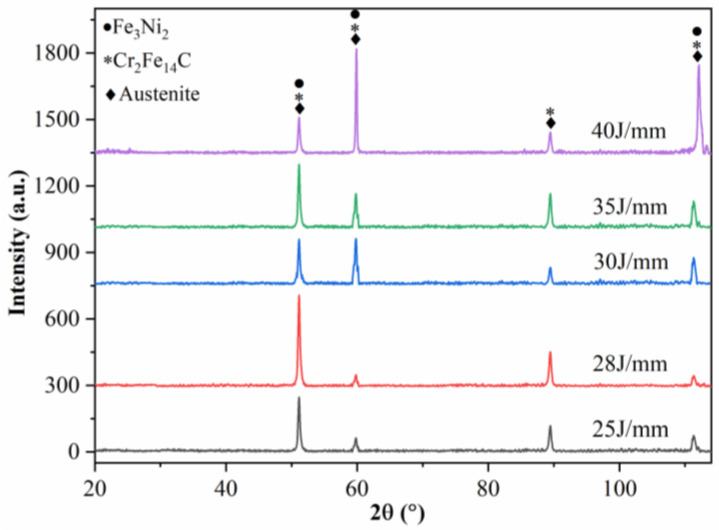
XRD images at different heat inputs.

X-ray diffraction analysis was conducted to ascertain the phase structure within the weld, with the findings presented in Figure 9. The diffraction peaks were appropriately calibrated, revealing the presence of austenite, Cr_2_Fe_14_C, and Fe_3_Ni_2_ as the characteristic peaks. In light of the aforementioned analysis, it can be inferred that the phases precipitated outside the grain consist of Cr_2_Fe_14_C and Fe_3_Ni_2_.

### 3.3. Performance Evaluation

**Figure 10:**
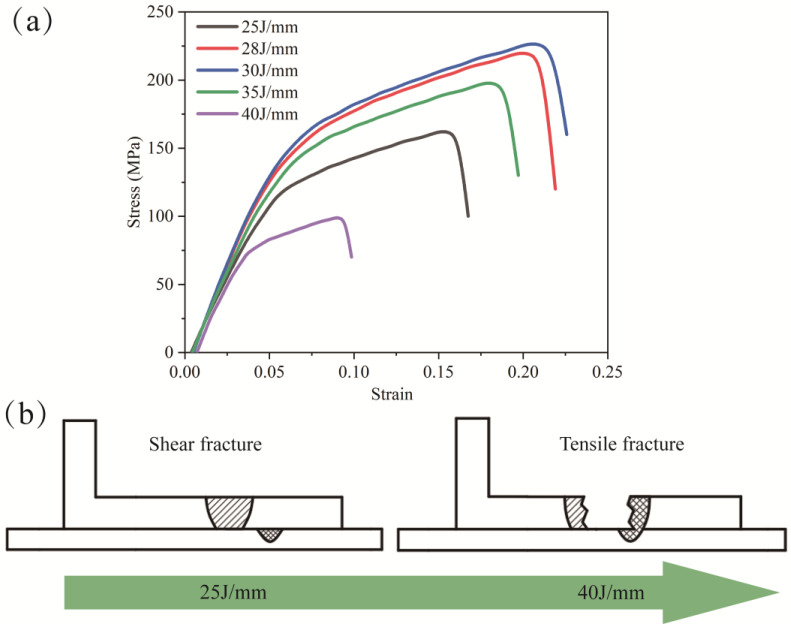
(**a**) Tensile shear strength with different heat inputs; (**b**) Mode of fracture in welding.

In the experimental procedure, tensile-shear testing is conducted at room temperature using an electronic universal tensile testing machine. The overlay welding method is employed, and corresponding gaskets are incorporated during the test to ensure coaxial tension [22]. Figure 10 illustrates the maximum stress of the joints under varying heat inputs. It is observed that when the heat input ranges from 25 to 30 J/mm, shear fracture takes place at the interface of dissimilar materials. The corresponding tensile strengths are measured as 162 MPa, 220 MPa, and 226 MPa. Conversely, when the heat input is 35 J/mm and 40 J/mm, tensile fracture occurs, resulting in tensile strengths of 198 MPa and 99 MPa, respectively.

Shear fracture is observed at heat inputs of 25 J/mm and 28 J/mm due to the narrow width of the bonding surface and the inability of the joint to withstand shear forces. At a heat input of 30 J/mm, there is a slight increase in the width of the melting zone. The presence of microcracks in the weld does not sufficiently weaken the tensile resistance, resulting in a shear fracture with improved strength. When the heat input exceeds 30 J/mm, the width of the bonding surface increases, leading to a significant increase in shear resistance and a shift in the fracture mode. However, as a result of heightened heat input, there is a phenomenon of element segregation taking place at the central region of the weld. The weld fractures when the thermal stress surpasses the stress threshold at the focal point of the weld joint. When subjected to a tensile force, the tensile resistance of a material is diminished as a result of the presence of cracks.

**Figure 11:**
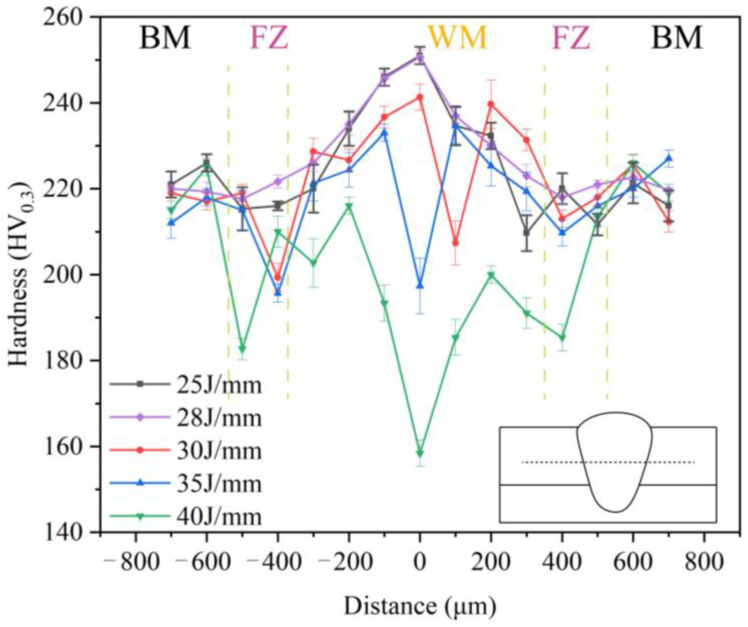
Distribution of microhardness of joints under different heat inputs.

The hardness of a material is influenced by various factors, including grain size, metallurgical changes, and alloy formation. The finer the grain size, the higher the hardness of the material. In the process of laser welding, alterations in the amount of heat applied can lead to metallurgical transformations, specifically the occurrence of martensitic phase transitions. The incorporation of alloy elements has the potential to alter the mechanical characteristics of a material. For instance, the inclusion of elements like nickel (Ni) and chromium (Cr) has been found to enhance the hardness of the weld. Through the analysis of the phase, it has been determined that the weld seam remains in the austenitic state and no significant metallurgical alterations have taken place. Additionally, the composition of the weld element has been identified. Consequently, this article excludes the examination of the impact of metallurgical changes and the formation of alloys on the performance of the weld. Figure 11 illustrates the distribution of microhardness perpendicular to the weld direction at various heat inputs, with the measurement point located 0.4 mm above the material interface. The figure reveals a significant variation in hardness values within the weld. Specifically, when the heat input is 25 J/mm, the average microhardness of the joint is 224 HV_0.3_. Notably, the hardness value in the middle of the weld is considerably higher than that of the base material, while the hardness value at the fusion line is the lowest. This observation aligns with the Hall–Page fine grain strengthening theory [23], which suggests that the center of the weld spontaneously nucleates to form a fine equiaxed crystal. As a result, the grain boundary area occupies a relatively large portion, requiring a greater load for crystal slip, thereby resulting in higher weld hardness. Conversely, the presence of soft areas at the fusion line leads to a lower intergranular dislocation energy. Upon increasing the heat input to 28 J/mm, the hardness of the weld center remains unchanged, but the hardness of the fusion zone decreases. Subsequently, when the heat input is further increased to 40 J/mm, the microhardness within the weld experiences a significant decrease. The average microhardness decreases to only 195 HV_0.3_, with certain positions exhibiting hardness values lower than that of the base metal. The cooling process of the weld seam is extended due to a significant heat input, leading to a decrease in the rate of cooling and an increase in brittleness. Additionally, the extended exposure of the weld to high temperatures leads to grain growth and a subsequent reduction in hardness. The precipitation of numerous carbides within the weld further exacerbates the situation, resulting in cracks under welding stress and a significant reduction in microhardness. Consequently, when the heat input is set at 25 J/mm, the weld strength is optimal.

**Figure 12:**
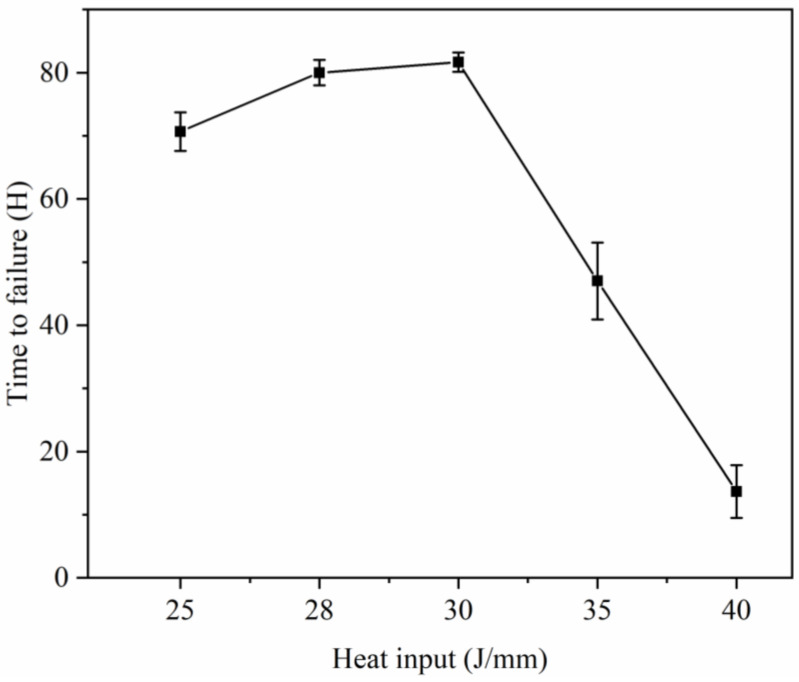
Failure time at different heat inputs.

Figure 12 illustrates the failure time of welded joints subjected to various heat inputs during the impact test. The figure illustrates that the highest level of temperature shock resistance and the longest failure time of 80 h are observed when the heat input is 30 J/mm. However, as the heat input increases further, reaching 40 J/mm, the impact resistance time decreases significantly to only 15 h. An examination of the curve slope K indicates that when the heat input reaches 28 J/mm, K = 3.1, signifying a substantial enhancement in temperature shock ability. In the range of 28–30 J/mm, K = 0.8, indicating a relatively insignificant change. Beyond 30 J/mm, K = −8, indicating a significant reduction. Consequently, the optimal temperature shock resistance of the joint is achieved when the heat input ranges from 28–30 J/mm.

## 4. Conclusions

1. The heat input above 25 J/mm can break through the contact thermal resistance of 1 mm stainless-steel- and nickel-based alloy 601 and form an effective molten pool. The penetration width and penetration depth of the weld increase with the increase of heat input. At the same time, the crack defects in the molten pool are becoming more and more significant.
2. The proportion of nickel-based alloys in the weld is observed to rise as the heat input increases. This rise in heat input leads to alterations in the elemental content of the weld, resulting in the presence of a significant quantity of chromium (Cr) and nickel (Ni) elements outside the grain at the center of the weld. Analysis of the phase structure composition through X-ray diffraction (XRD) reveals the presence of abundant Cr_2_Fe_14_C and Fe_3_Ni_2_ phases.
3. In instances of low heat input, the joint exhibits shear breakage. As the heat input increases, the width of melting also increases, leading to tensile fracture. However, the tensile fracture strength is compromised due to the presence of longitudinal cracks in the center. When the heat input is below 40 J/mm, the weld’s Vickers hardness surpasses that of the base metal. Conversely, when the heat input reaches 40 J/mm, the weld’s Vickers hardness is lower than that of the base metal.
4. With the escalation of heat input, there is a notable decrease in the joint’s ability to withstand temperature shocks. The joint exhibits its highest capacity when subjected to a heat input range of 28–30 J/mm. In order to enhance the joint’s ability to withstand temperature shocks, the utilization of high heat input is circumvented.

## Figures and Tables

**Table 1:** Main chemical composition of materials (wt%).

	Fe	Cr	Ni	Mn	Si	C	Al	Ti	Mo	N	S	P
316L	69.789	16.32	10.11	1.11	0.51	0.016	-	-	2.06	0.032	0.001	0.022
Inconel 601	14.05	23.14	59.57	0.72	0.33	0.05	1.46	0.44	-	-	0.001	0.02

**Table 2:** Laser welding parameters.

Experiment Serial Number	Laser Power (W)	Welding Speed (mm/s)	Heat Input (J/mm)
1	200	8	25
2	196	7	28
3	240	8	30
4	210	6	35
5	240	6	40

**Table 3:** Elemental content inside and outside the grain.

Heat Input (J/mm)	Scan Location	Average Content of Chemical Elements (wt%)			
		C	Cr	Fe	Ni
25	Inside the grain	3.84	16.30	60.30	15.39
	Outside the grain	3.95	16.38	59.69	16.38
28	Inside the grain	4.08	15.88	65.09	11.53
	Outside the grain	4.44	16.48	64.07	12.86
30	Inside the grain	4.10	15.94	62.37	12.23
	Outside the grain	4.39	16.55	61.05	13.61
35	Inside the grain	4.99	16.04	53.96	23.58
	Outside the grain	5.27	17.12	50.8	24.09
40	Inside the grain	4.57	16.39	56.07	20.42
	Outside the grain	5.14	17.82	51.61	24.85

## Data Availability

The research data in question pertains to sensitive company information and is not suitable for public disclosure.
